# A Nickel Dissolution Process for Multilayer Electroforming to Achieve Ultrahigh Adhesion Strength

**DOI:** 10.3390/ma16196504

**Published:** 2023-09-30

**Authors:** Zhuangzhuang Wang, Chunjian Shen, Zhou Ma, Zengwei Zhu, Di Zhu

**Affiliations:** College of Mechanical and Electrical Engineering, Nanjing University of Aeronautics and Astronautics, Yudao Street 29, Nanjing 210016, China; 18351952596@163.com (Z.W.); shencj@nuaa.edu.cn (C.S.); mazhounuaa@163.com (Z.M.); dzhu@nuaa.edu.cn (D.Z.)

**Keywords:** multilayer electroforming, adhesion performance, nickel, dissolution efficiency

## Abstract

Multilayer electroforming has a high potential to produce Ni/Ni layer structured metal walls with excellent material properties and a high thickness uniformity. However, Ni is easily oxidized in air, which fundamentally leads to a low adhesion strength between the Ni layers. Here, a novel in situ treatment is proposed for improving the adhesion performance between Ni layers. This treatment integrated the steps of electrochemical dissolution, surface protection, and electroforming. A study of the polarization behavior implied the electroformed Ni layer was dissolved efficiently in the NH_2_SO_3_H solution, beginning at a dissolution current density of 5 A·cm^−2^, which could remove the oxide film. A smooth substrate surface with a good surface hydrophilicity was obtained starting at 8 A·cm^−2^, helping to protect the activated substrate from being contaminated and oxidized. The experimental results showed that ultrahigh normal and shear adhesion strengths over 400 MPa between the Ni layers were achieved.

## 1. Introduction

Multilayered metal materials have attracted extensive attention for their potential in achieving excellent performances. It has been revealed that a Cu/Cu multilayered structure can effectively improve the toughness and fatigue strength of Cu [1]. Thin Zr/Nb NMMs exhibit a higher radiation resistance and lower irradiation hardening, which are promising candidates for nuclear applications [2]. An extraordinarily high yield strength has been achieved in a multilayered Ti/Ti structure, which also possesses an excellent combination of a fine-grain strength and coarse-grain ductility [3]. Through designing the thickness ratios of Cu and Ni, Cu/Ni laminated composites can achieve an ultrahigh strength [4,5]. These multilayer structures were prepared by cold rolling and annealing processes, which are convenient for producing large sheets, but inconvenient for producing complex-shaped thin-wall parts, especially thin-walled revolving parts with a complex profile.

Electroformed Ni is widely applied as walls in parts of thrust chambers of liquid rocket engines, liners, storage tanks of nuclear fuel, wind tunnel nozzles, and so on [6,7]. Normally, these electroformed Ni walls have a complex geometry and large wall thickness, and their material properties and thickness distribution are the key parameters [8,9,10]. For improving these material properties, some researchers have added special chemical additives to the electroforming bath, but these additives tend to decompose and even absorb on the cathode surface, leading to co-deposition with metal [11,12]. Then, some researchers have carried out cathode polishing during the electroforming process, such as brush-assisted polishing and particle-assisted polishing [13,14], obtaining Ni with good material properties. For improving the thickness distribution, some researchers have optimized the anode geometry, added auxiliary electrodes, or shielded the local electric field to uniform the cathode electric field distribution [15]. However, as deposits grow thicker to change the cathode geometry, the cathode electric field distribution becomes ununiform again. Up to now, there are no effective methods for improving the uniformity of electroformed Ni with a large thickness.

Multilayer electroforming has a high potential to obtain multilayered Ni/Ni structures not only with good material properties, but also with a uniform thickness distribution if the adhesion strength between the electroformed layers is high enough [16,17,18]. Ni is easily oxidized in air, which leads to forming a dense oxide film on the surface. This oxide film is the primary cause of the low adhesion strengths between the electroformed Ni layers. Moreover, it has a high corrosion resistance and is hard to remove using strong acid etching or even anode electrolyzation [19,20]. Only one report successfully increased the adhesion strength between Ni/Ni multilayers in the MEMS field by applying a unique pulse reverse current technique to the nickel chloride plating solution [18]. The Ni oxide film was removed in the positive pulse and the Ni layer was electrodeposited in the negative pulse, resulting in high adhesion strengths. However, the pulse reverse current technique is only suitable on the microscale, where the oxide film is thin. It demonstrates that removing the oxide film and electroforming on an active surface are necessary conditions for achieving a high adhesion strength between electroformed Ni layers.

**Figure 1:**
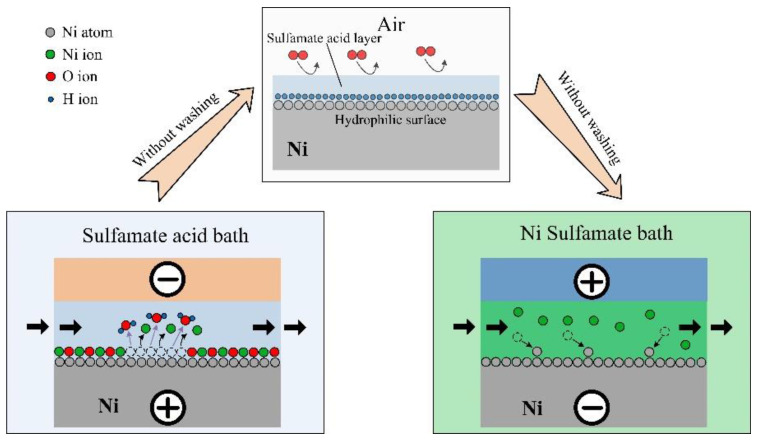
Diagram of the in situ-treatment.

In this paper, we found that electroformed Ni layers have a high dissolution efficiency in acid sulfamate baths. Sulfamate baths are commonly used for Ni electroforming. Thus, an in situ treatment was performed to improve the adhesion performance between the Ni layers in a sulfamic acid electrolyte. This treatment integrated electrochemical dissolution, surface protection, and electroforming, as shown in Figure 1. Electrochemical dissolution was applied to remove the Ni oxide film and achieve an active, hydrophilic surface. Then, a sulfamic acid layer was adsorbed on the activated surface to protect the active surface from being contaminated and oxidized during the substrate transport from the sulfamate acid bath to the Ni sulfamate bath. At last, the Ni layers were electroformed on the active substrate. Both the normal and shear adhesion strengths between the Ni layers were studied to comprehensively analyze the adhesion performance.

## 2. Materials and Methods

### 2.1. Polarization and Methods

Linear sweep voltammetry was measured to obtain anodic polarization curves of the electroformed Ni in a 1.2 M NH_2_SO_3_H (Macklin, Shanghai, China) solution and 0.5 M H_2_SO_4_ (Macklin, Shanghai, China) solution. It was carried out using an electrochemical workstation (Zennium E, Zahner, Kronach, Germany) with a modified three-electrode system. A calomel electrode and platinum electrode were used as the reference electrode (RE) and counter electrode (CE), respectively. An electroformed Ni sheet specimen of 5 mm × 5 mm × 10 mm was used as the working electrode (WE). The scan rate of the polarization curve was 1 mV∙s^−1^ and the scan range was from −2 V to 4 V.

### 2.2. Dissolution Experiments

The anodic reaction mainly includes two reactions; the primary reaction for metal dissolution is:

Ni → Ni^+^ + e^−^ (1)

and due to the oxide film on the surface, an oxygen evolution will simultaneously occur as the secondary reaction:

2H_2_O → O_2_ + 4H^+^ + 4e^−^ (2)

**Figure 2:**
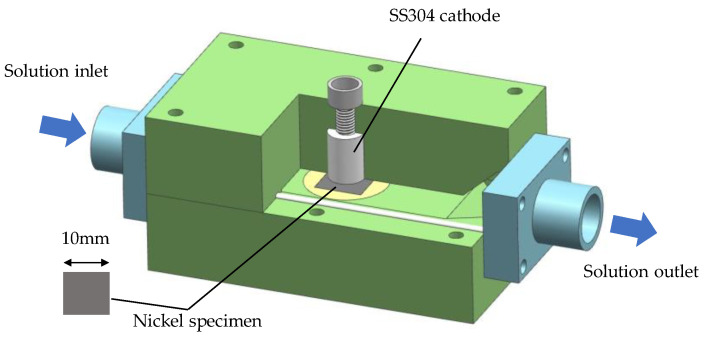
Diagram of the experimental set-up for dissolution experiments.

For investigating the current efficiency, an electroformed Ni sheet specimen of 10 mm × 10 mm × 10 mm was dissolved with different current densities. The experimental set-up is shown in Figure 2. The cathode was made of stainless steel 304. The inter-electrode gap was 2 mm. The measurements of the current efficiency were based on the weight loss of the Ni sheet specimen, which was weighed by analytical balance (JT3003D, LICHEN, Quanzhou, China), and the current efficiency was described as follows:

(3) η=M/KIt

where *M* is the actual mass (g) dissolved from the anode by a current *I* (A) passing for time *t* (s), *η* is the current efficiency during the dissolution, and *K* is the theoretical mass electrochemical equivalent (g·(A∙s)^−1^).

### 2.3. Normal Adhesion Strength Test

**Figure 3:**
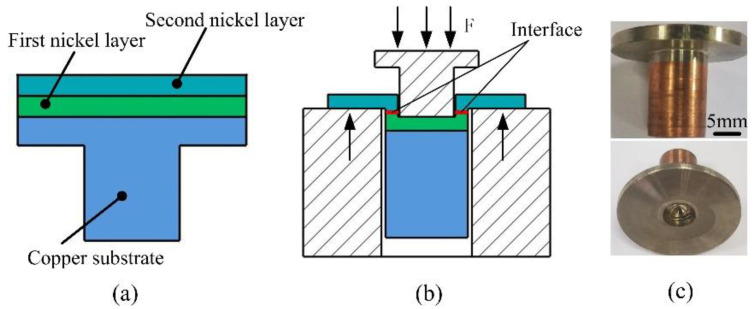
Diagram of the Ollard method: (**a**) composition of test piece; (**b**) test process; and (**c**) image of test piece.

The Ollard method was used to test the normal adhesion strength between the Ni layers [21]. As is shown in Figure 3a, the first Ni layer and second Ni layer were electroformed on a copper substrate successively. An in situ treatment was carried out between the first and second Ni layers using the set-up shown in Figure 2. Figure 3b shows the normal adhesion strength *σ* (MPa) test process of the Ollard method, and it is described as follows:

(4) σ=F/S

where *F* is the loading force (N) and *S* is the area (mm^2^) of the interface between the first and second Ni layers. Figure 3c shows images of the test piece.

### 2.4. Shear Adhesion Strength

**Figure 4:**
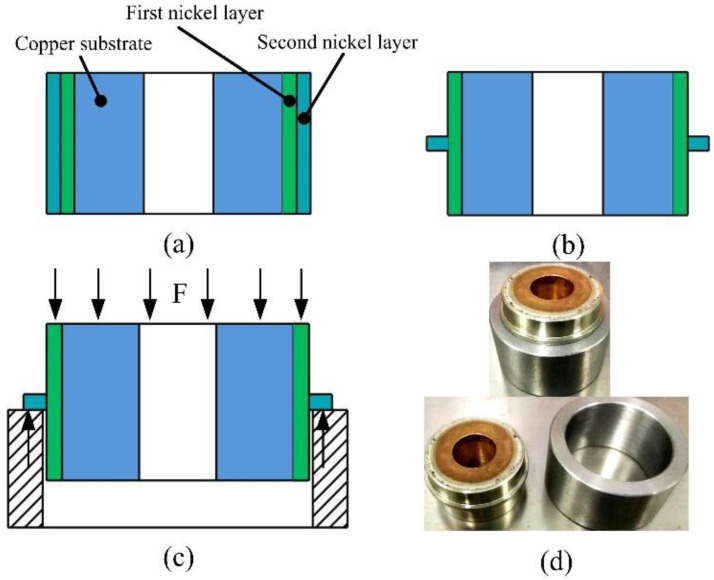
Diagram of the ring shear method: (**a**) composition of test piece; (**b**) the piece after processing; (**c**) test process; and (**d**) image of test piece.

**Figure 5:**
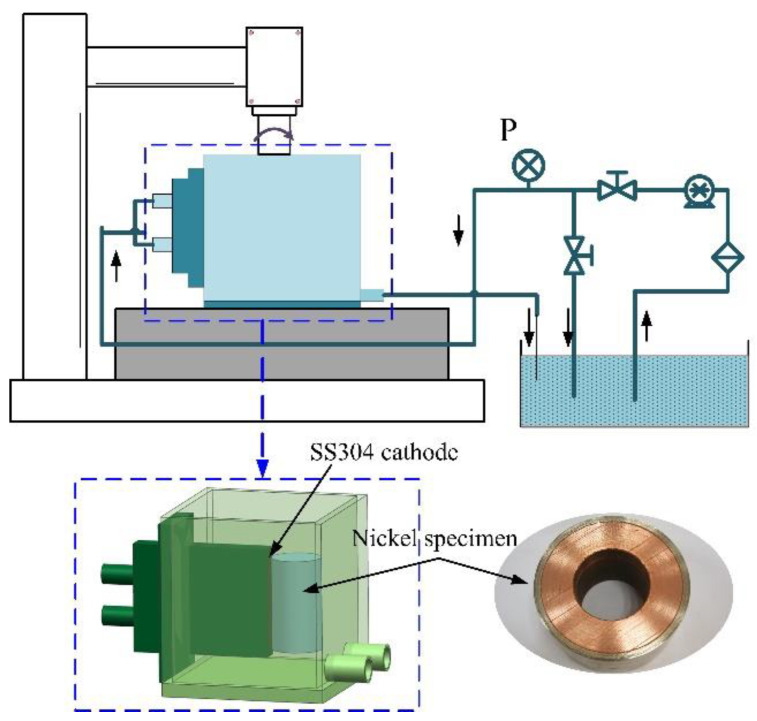
Diagram of the in situ treatment set-up for rotation specimen.

The ring shear method was used to test the shear adhesion strength between the Ni layers. As shown in Figure 4, a rotation specimen of Ø35 mm × 20 mm was used as a sample for the shear adhesion strength test. As seen, the first and second Ni layers were electroformed on a copper rotation substrate successively. The in situ treatment process was also carried out between the first and second Ni layers. Figure 4c shows the shear adhesion strength test process, and it includes the ratio of the loading force (N) and the area (mm^2^) of the interface. Figure 4d shows images of the test specimen. A special device was designed to carried out the in situ treatment, as shown in Figure 5. The rotation specimen was revolved and a narrow sheet cathode was placed beside it with an inter-electrode gap of 2 mm. The scanning speed was determined by the rotation speed. When a direct voltage was applied with the electrolyte flushing the surface, the materials of the rotation specimen were dissolved electrochemically. The normal adhesion strength test and shear adhesion strength test were carried out in universal testing machine (CMT6103, MTS, Shanghai, China).

## 3. Results and Discussion

**Figure 6:**
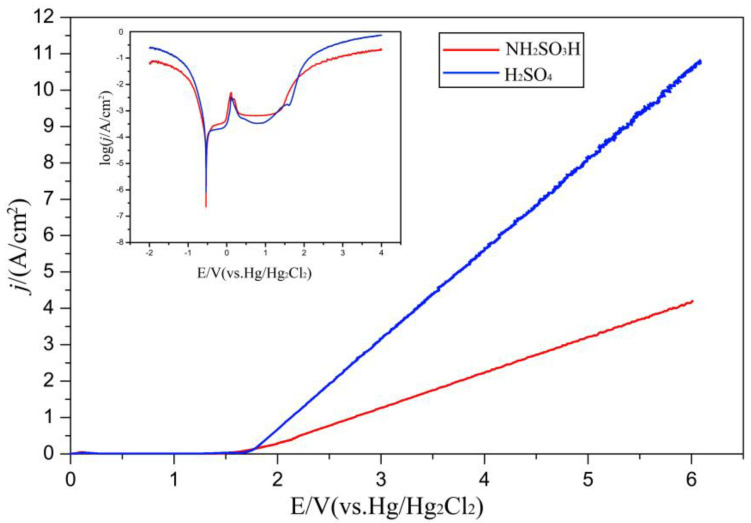
Polarization curves of Ni layer in NH_2_SO_3_H solution and H_2_SO_4_ solution.

Figure 6 shows the polarization curves of the Ni layer in a 1.2 M NH_2_SO_3_H solution and 0.5 M H_2_SO_4_ solution, respectively. The influence of the electrolyte on the dissolution behavior of the Ni layer was analyzed. As can be seen, the polarization curve in the NH_2_SO_3_H solution showed active, passive, and transpassive transition behaviors. The passive region was from 0.03 V to 1.41 V, where the current no longer increased with the potential. When the potential exceeded 1.41 V, the current density increased sharply. This may have been because the Ni layer began to dissolve, or the oxygen evolution happened on the surface of the Ni layer. For the H_2_SO_4_ solution, the polarization curve showed a different feature: it consisted of active, first passive, second passive, and transpassive regions. The first passivation occurred at 0.03 V, and the second passivation occurred at 1.68 V. The reason for two passivation regions being in the curve may have been that the Ni layer was immediately oxidized in the H_2_SO_4_ solution at the beginning, forming a passivation film with defects or pores on the surface. When the potential increased to 0.9 V, breakages of the passivation films occurred at the area of defects or pores. Thus, some areas of the Ni layer were exposed to the acid solution and reacted again. Then, the current increased. However, new passive films were formed rapidly at the broken area. This would hinder the Ni layer from being further dissolved, showing the second passivation behavior [22]. When the potential exceeded 1.73 V, the current increased again and an ohmic behavior occurred, indicating that the Ni layer began to dissolve or the oxygen evolution happened. However, in the NH_2_SO_3_H solution, Ni ions and sulfamic ions could form a metal complex, which would not form a continuous sulfamic acid layer on the dissolved surface and could protect the surface from oxidation.

**Figure 7:**
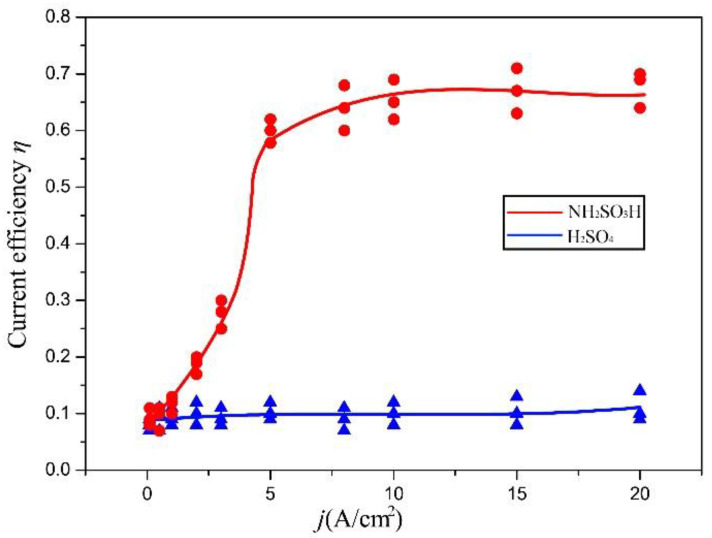
η–j curves of Ni layer in NH_2_SO_3_H solution and H_2_SO_4_ solution.

Plots of the η–j curves of the Ni layer in the NH_2_SO_3_H solution and H_2_SO_4_ solution are displayed in Figure 7, and the values of the dissolution current density ranged from 0 to 20 A·cm^−2^. For the NH_2_SO_3_H solution, there was a high dependence of the current efficiency on the value of the dissolution current density. When the dissolution current density was 1 A·cm^−2^, the current efficiency was around 10%. Then, the current efficiency would increase to a high value of about 65% when increasing the dissolution current density to 5 A·cm^−2^. After 5 A·cm^−2^, the current efficiency was stable at around 65%, even if the current density continued to increase. This meant the Ni layer could be dissolved in the NH_2_SO_3_H solution beginning at a dissolution current density of 5 A·cm^−2^. For the H_2_SO_4_ solution, the η–j curve showed many differences. The current efficiency was always around 10%, which almost did not change with an increase in the dissolution current density. This implies that the Ni layer had a strong passivation characteristic in the H_2_SO_4_ solution. Combining the polarization curves and η–j curves for analysis, the increase in the current density in the transpassive regions was mainly due to the dissolution of the Ni layer in the NH_2_SO_3_H solution, and due to the oxygen evolution in the H_2_SO_4_ solution. Thus, the NH_2_SO_3_H solution was selected to dissolve the Ni layer in the step of the electrochemical dissolution to remove the surface oxide film, and the dissolution current density should start at 5 A·cm^−2^.

**Figure 8:**
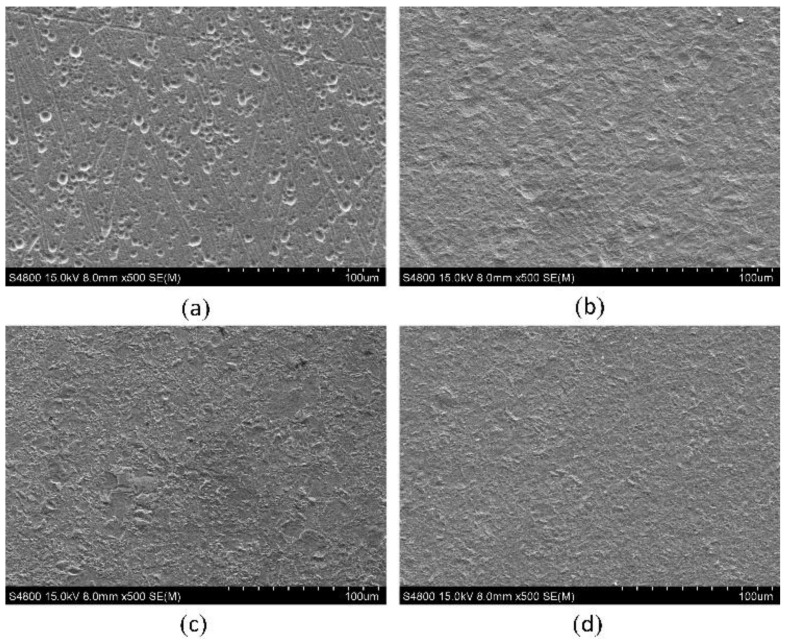
Micromorphology of Ni layer dissolved with different dissolution current densities: (**a**) 2 A·cm^−2^; (**b**) 5 A·cm^−2^; (**c**) 8 A·cm^−2^; and (**d**) 10 A·cm^−2^.

Figure 8 shows the surface micromorphology of the Ni layers dissolved with different dissolution current densities in the NH_2_SO_3_H solution. The surface micromorphology was evaluated using scanning electron microscopy (SEM, Sigma 300, Oberkochen, Germany). As can be seen, the surface smoothness of the Ni layer improved with an increase in the dissolution current density. When the dissolution current density was 2 A·cm^−2^, dense pits appeared on the surface of the Ni layer, together with the initial surface texture from polishing. This means that selective corrosion occurred, and some areas were protected by the passive films. This is in accordance with a low current efficiency of about 12% at 2 A·cm^−2^. When the dissolution current density was increased to 5 A·cm^−2^, the surface of the Ni layer was corroded overall. However, the surface quality was low due to many apparent micro-pits. When the dissolution current density was 8 A·cm^−2^, the micro-pits disappeared, but a large number of micro-protrusions appeared on the surface. With further increasing the dissolution current density to 10 A·cm^−2^, the height of the micro-protrusions decreased. It can be assumed that, if an appropriate dissolution current density between 5 A·cm^−2^ and 8 A·cm^−2^ was adopted, the surface of the Ni layer would be smoother.

**Figure 9:**
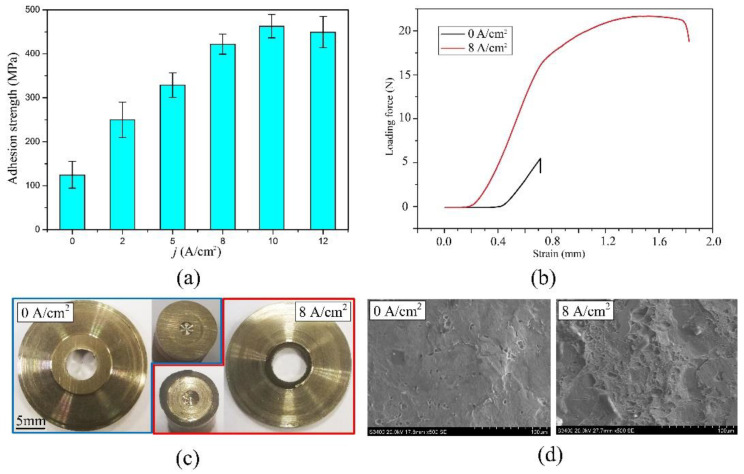
Test results of the normal adhesion strength between the Ni layers: (**a**) test curves; (**b**) adhesion strength; (**c**) macro images of fracture surface; and (**d**) SEM images of fracture surface.

**Figure 10:**
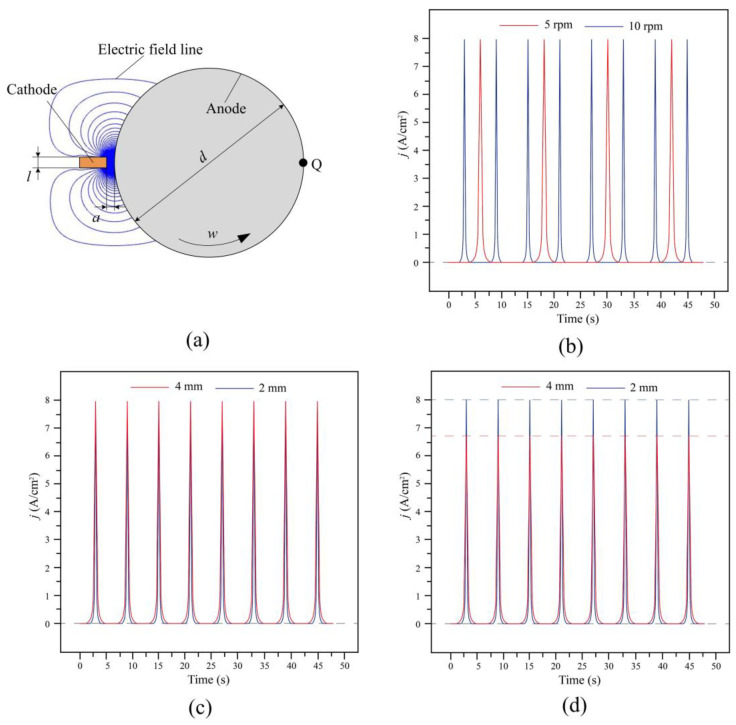
Change of dissolution current density at a certain point with the time: (**a**) simulation model; (**b**) different ration speed w; (**c**) different cathode width l; and (**d**) different inter-electrode gap a.

Figure 9 shows the test results of the normal adhesion strength between the Ni layers. As shown in Figure 10a, the normal adhesion strength between the Ni layers improved with an increase in the dissolution current density from 0 A·cm^−2^ to 8 A·cm^−2^, and then it stayed around 430 MPa after 8 A·cm^−2^. As shown in Figure 8, at a low dissolution current density, only selective corrosion occurred on the surface of the Ni layer. Thus, the oxide film could not be removed effectively, resulting in a low normal adhesion strength between the Ni layers. With increasing the dissolution current density, the current efficiency of the Ni layer increased according to Figure 7. That means the dissolution area on the Ni layer increased so that the area of the oxide film decreased, resulting in an increase in the normal adhesion strength. Above 5 A·cm^−2^, the current efficiency was stable at around 65%. The most likely reason for the increase in the normal adhesion strength is that the grain boundaries and grains were exposed on the surface due to removing the oxide film, and, in this condition, the bond between two layers was mainly a metallic bond. After 8 A·cm^−2^, the oxide film was removed effectively. Thus, the normal adhesion strength was stable at around 430 MPa. To sum up, in order to achieve a high normal adhesion strength between Ni layers, the dissolution current density should start at 8 A·cm^−2^. Figure 9b shows the test curves of the test pieces prepared without and with the in situ treatment, in which the dissolution current density was 8 A·cm^−2^. Because of the uneven surface of the test piece, there was always a detection distance where the force was zero. These data were retained, and it was easy to observe the differences between two curves in this situation. As is shown, the test curve of the pieces without the in situ treatment only had an elastic deformation region, while the test curve of the pieces with the in situ treatment had both an elastic deformation region and a plastic deformation region. The images of the fracture surface are in accordance with the test curves, as shown in Figure 9c,d. The fracture surface of the test piece without the in situ treatment was smooth, and SEM images show there were few dimples. This indicates the fracture happened at the interface. The test pieces with the in situ treatment at a dissolution current density of 8 A·cm^−2^ had obvious torn metallic zones on the fracture surface. SEM images show there were lots of dense dimples. This means the fracture happened inside the Ni layer after the in situ treatment. The connections between two electrodeposition layers included mechanical connections, physical connections, and electrochemical connections. Electrochemical connections mean different layers connected by metallic bonds. According to the microtopography of the fracture surface, the connection between the layers was a metallic bond and it resulted in an ultrahigh normal adhesion strength.

In order to comprehensively analyze the adhesion performance, the shear adhesion strength between the Ni layers was studied. A rotation specimen was used as sample for the shear adhesion strength test. As shown in Figure 4, a special device was designed to carry out the in situ treatment on the rotation specimen. During the electrochemical dissolution process, the dissolution position on the surface changed periodically with the rotation. The change in the current density at a certain point with the time was simulated using COMSOL. Table 1 shows the simulation parameters. Figure 10 shows the simulation results. As is shown, the material dissolution at a certain point Q presented a pulse characteristic, where the wave of the dissolution current density distribution was sub-triangular. Figure 10b shows that the pulse period and rising time of the dissolution current density were inversely proportional to the rotation speed. Figure 10c,d shows the pulse period and rising time of the dissolution current density were proportional to both the cathode width and the inter-electrode gap.

**Figure 11:**
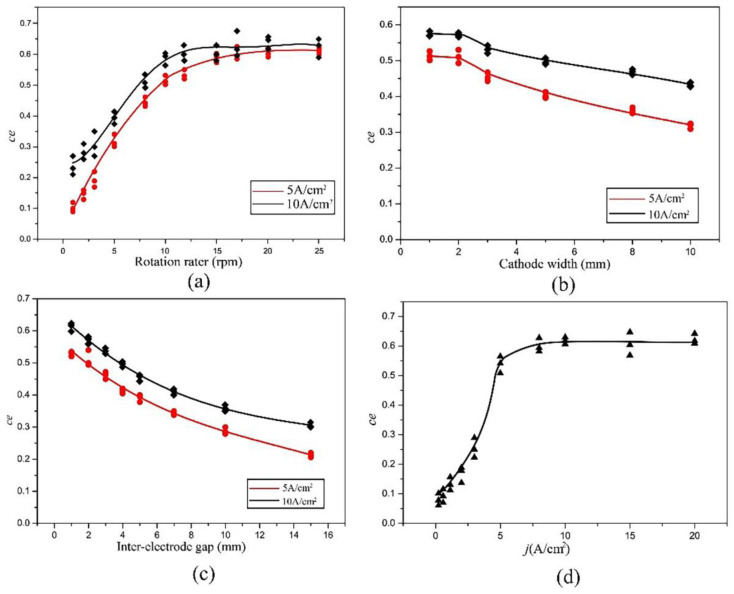
Plots of the current efficiency (*ce*) curves of the Ni rotation specimen: (**a**) with the ration speed; (**b**) with the cathode width; (**c**) with the inter-electrode gap; and (**d**) with the peak dissolution current density.

**Figure 12:**
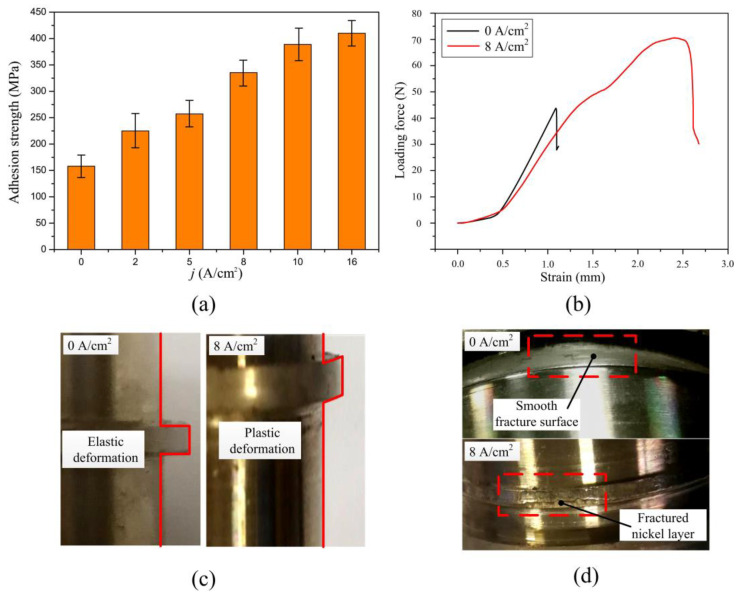
Test results of the shear adhesion strength between the Ni layers of the rotation specimen: (**a**) change of the adhesion strength with the peak current density; (**b**) test curves; (**c**) images of the test specimen after the force unloading; and (**d**) macro images of fracture surface.

Plots of the current efficiency (ce) curves of the Ni rotation specimen in the NH_2_SO_3_H solution with different rotation speeds, cathode widths, and inter-electrode gaps were measured. The values of the rotation speed, cathode width, and inter-electrode gap ranged from 0 rpm to 25 rpm, 0 mm to 10 mm, and 0 mm to 15 mm, respectively. For the rotation speed in Figure 11a, whether the peak dissolution current density was 5 A·cm^−2^ or 10 A·cm^−2^, there was a high dependence of the current efficiency on the rotation speed when the speed was less than 10 rpm. This dependence disappeared when the speed was over 10 rpm. This was due to the loss of the current efficiency in the low dissolution current density period at the rising and falling edge according to Figure 7. With increasing the rotation speed, the rising and falling time of the dissolution current density decreased, meaning the period of low dissolution current density was reduced. If the rotation speed was high enough, the rising and falling times of the dissolution current density were almost zero, so that there was no low current density period. This is why the rotation specimen with a rotation speed of 10 rpm had a similar current efficiency to the Ni sheet specimen at the current density of 10 A·cm^−2^. For the cathode width in Figure 11b, the current efficiency was similar at the cathode widths of 1 mm and 2 mm, and then it decreased with an increase in the inter-electrode gap, starting at 2 mm. This is because the increase in the cathode width expended the low dissolution current density period at the rising and falling edge shown in Figure 11b. Then, the increase in the inter-electrode gap also expended the low dissolution current density period at the rising and falling edge shown in Figure 11c. Thus, the current efficiency decreased with an increase in the inter-electrode gap. From the polarization curves of the Ni layer, we found that only when the current density was above 5 A·cm^−2^ could Ni dissolve effectively in the sulfamic solution. According to the simulation result, the rotation speed, cathode width, and inter-electrode gap would affect the period of the current density, which would affect the dissolution of Nickel. In conclusion, the rotation speed, cathode width, and inter-electrode gap greatly affected the current efficiency during the electrochemical dissolution on the whole surface. All of them should be optimized to obtain a high current efficiency. Here, according to Figure 11, a ration speed of 10 rpm, a cathode width of 2 mm, and an inter-electrode gap of 2 mm were chosen. Based on this, the η–j curve of the Ni rotation specimen was measured and is shown in Figure 11d, where j is the peak dissolution current density. The values of the peak dissolution current density ranged from 0 A·cm^−2^ to 20 A·cm^−2^. As can be seen, the change in the current efficiency with the peak dissolution current density was similar to that of the Ni sheet specimen in the NH_2_SO_3_H solution. The current efficiency increased to a high value of about 60% when the peak dissolution current density increased to 5 A·cm^−2^, and it remained stable around 60%, even if the peak dissolution current density continues to increase. According to Figure 7 and Figure 12, it was implied that the current efficiency of the rotation specimen was similar to the sheet specimen after optimizing the rotation speed, the cathode width, and the inter-electrode gap. Thus, the Ni surface oxide film of the rotation specimen could be dissolved efficiently.

Figure 12 shows the test results of the shear adhesion strength. As can be seen in Figure 12a, the shear adhesion strength between the Ni layers improved with an increase in the peak dissolution current density, which was the same as the test results of the normal adhesion strength. Figure 12b shows the test curves of the test pieces prepared without the in situ treatment and with the in situ treatment, in which the peak dissolution current density was 8 A·cm^−2^. As is shown, the test curve of the pieces without the in situ treatment only had an elastic deformation region, while the test curve of the pieces with the in situ treatment had both an elastic deformation region and a plastic deformation region. Figure 12c shows the images of the test pieces unloaded before they were fractured. The test pieces restored and deformed after the force unloading without and with the in situ treatment. Figure 12d shows the macro fracture surface of the test pieces. As can be seen, the fracture surface of the test piece without the in situ treatment was smooth, which indicates the fracture happened at the interface, while the fracture surface of the test piece with the in situ treatment had obvious torn metallic zones, indicating the fracture happened inside the Ni layer. This implies the shear adhesion strength between the Ni layers greatly improved after the in situ treatment, which was almost the same as the tensile strength of the Ni layers.

**Figure 13:**
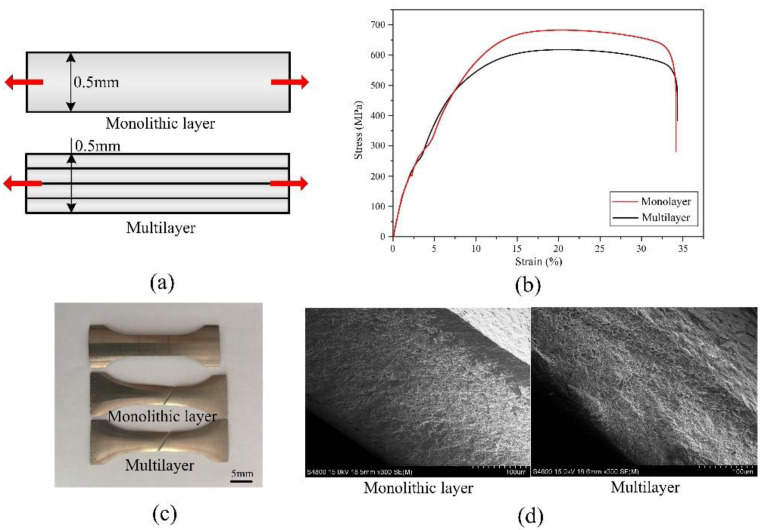
Test results of the Ni monolayer and the Ni/Ni multilayer: (**a**) structure of test piece, (**b**) test curve, (**c**) macro images of fracture surface; and (**d**) SEM images of fracture surface.

After improving both the normal and shear adhesion strength between the Ni layers, the Ni/Ni multilayer was electroformed to analyze its performance. Figure 13 shows the results of the tensile strength test of a monolithic Ni layer obtained via one-time electroforming and a Ni/Ni multilayer obtained via multilayer electroforming. As shown in Figure 13a, the monolithic Ni layer was 0.5 mm thick, and the Ni/Ni multilayer, which had four layers, was also 0.5 mm thick. Figure 13b shows the test curves of the tensile strength test. As can be seen, both of them exhibited good mechanical properties. The tensile strength of the monolithic Ni layer was slightly above that of the Ni/Ni multilayer, which were 682 MPa and 618 MPa, respectively. They both showed excellent elongations of 33% and 34%, respectively. After the tensile strength test, obvious necking at the fracture was observed at both of the monolithic Ni layer and the Ni/Ni multilayer. Figure 13d shows SEM images of the micromorphology at the fracture surface. As is shown, both of them had dense dimples on the fracture surface, implying they had good mechanical properties. Moreover, there were no delamination and microcracks observed on the fracture surface of the Ni/Ni multilayer. If these microcracks exist, they would grow towards the layers and finally result in fatigue fracture. This means an excellent adhesion performance was achieved between the Ni layers and we can manufacture thick Ni parts with a uniform thickness and good mechanical properties using multilayer electroforming. In conclusion, the Ni/Ni multilayer manufactured via the in situ treatment process in this paper had similar mechanical properties with the monolithic Ni layer.

## 4. Conclusions

In this paper, an in situ treatment was carried out to improve the adhesion performance between Ni layers during multilayer electroforming. This treatment integrated the steps of electrochemical dissolution, surface protection, and electroforming in a sulfamic acid electrolyte. This could obtain and maintain an active ultraclean substrate for the growth of a Ni layer. The conclusions can be summarized as follows;

1. The electroformed Ni layer was dissolved with a current efficiency of about 65% in the NH_2_SO_3_H solution beginning at a dissolution current density of 5 A·cm^−2^. A smooth substrate surface with a good surface hydrophilicity was obtained starting at 8 A·cm^−2^.
2. Ultrahigh normal and shear adhesion strengths over 400 MPa between the Ni layers were achieved with the in situ treatment, in which the dissolution current density was 8 A·cm^−2^.
3. The Ni/Ni multilayer electroformed in this paper exhibited similar mechanical properties with the monolithic Ni layer obtained via one-time electroforming. Thus, multilayer electroforming with the in situ treatment has a high potential for manufacturing thick Ni walls with good properties, an excellent performance, and a uniform thickness.

## Figures and Tables

**Table 1:** Parameters of the simulation dissolution current density distribution.

Parameter	Value
Cathode current density (*j*)	8.5 A·cm^−2^
Cathode width (*l*)	2 mm, 4 mm
Diameter of specimen (*d*)	35 mm
Inter-electrode gap (a)	2 mm, 4 mm
Rotation speed (*w*)	5 rpm, 10 rpm
Conductivity (σ)	10 s·m^−1^

## Data Availability

All data can be obtained from the corresponding author upon reasonable request.

## References

[1] Liu H.S., Zhang B., Zhang G.P. (2011). Enhanced toughness and fatigue strength of cold roll bonded Cu/Cu laminated composites with mechanical contrast. Scr. Mater..

[2] Daghbouj N., Sen H.S., Čížek J., Lorinčík J., Karlík M., Callisti M., Čech J., Havránek V., Li B., Krsjak V. (2022). Characterizing heavy ions-irradiated Zr/Nb: Structure and mechanical properties. Mater. Des..

[3] Li D., Fan G., Huang X., Juul Jensen D., Miao K., Xu C., Geng L., Zhang Y., Yu T. (2021). Enhanced strength in pure Ti via design of alternating coarse- and fine-grain layers. Acta Mater..

[4] Tan H.F., Zhang B., Luo X.M., Zhu X.F., Zhang G.P. (2016). High-Cycle Fatigue Properties of Ultrafine-Scale Cu/Ni Laminated Composites. Adv. Eng. Mater..

[5] Liang F., Tan H., Zhang B., Zhang G. (2017). Maximizing necking-delayed fracture of sandwich-structured Ni/Cu/Ni composites. Scr. Mater..

[6] Mimura H., Matsuyama S., Sano Y., Yamauchi K. (2011). Surface replication with one-nanometer.level smoothness by a nickel electroforming process. Int. J. Electr. Mach..

[7] Li J., Qian J.-G., Hu X.-T., Li T.-J., Li H.-T. (2023). Microstructure and properties of electroformed nickel under different waveform conditions. Rare Met..

[8] Yang F., Li C., Cheng S., Wang L., Tian W. (2010). Microstructural evolution in electroformed nickel shaped-charge liners with nano-sized grains undergone deformation at ultrahigh strain rate. Int. J. Miner. Metall. Mater..

[9] Chu K., Bae K.D., Song B.G., Kim J., Park Y.Y., Xianyu W., Lee C.S., Sohn Y. (2018). Quantitative analysis of nano-defects in thin film encapsulation layer by Cu electrodeposition. Appl. Surf. Sci..

[10] Niu Y., Wei J., Zhao J., Hu J., Yu Z. (2013). Preparation and properties of nanosized multilayered Ni coatings by ultrasound-assisted electrodeposition. Acta Metall. Sin..

[11] Kang M., Gewirth A.A. (2003). Influence of additives on copper electrodeposition on physical vapor deposited (PVD) copper substrates. J. Electrochem. Soc..

[12] Abbott A.P., Ballantyne A., Harris R.C., Juma J.A., Ryder K.S. (2017). Bright metal coatings from sustainable electrolytes: The effect of molecular additives on electrodeposition of nickel from a deep eutectic solvent. Phys. Chem. Chem. Phys..

[13] Qian Y., Tan J., Liu Q., Yu H., Xing R., Yang H. (2011). Preparation, microstructure and sliding-wear characteristics of brush plated copper–nickel multilayer films. Surf. Coat. Technol..

[14] Lv B., Hu Z., Wang X., Xu B. (2015). Electrodeposition of nanocrystalline nickel assisted by flexible friction from an additive-free Watts bath. Surf. Coat. Technol..

[15] Volgin V.M., Lyubimov V.V., Gnidina I.V., Kabanova T.B., Davydov A.D. (2017). Effect of Anode Shape on Uniformity of Electrodeposition onto Resistive Substrates. Electrochim. Acta.

[16] Torabinejad V., Rouhaghdam A.S., Aliofkhazraei M., Allahyarzadeh M.H. (2016). Electrodeposition of Ni–Fe and Ni–Fe-(nano Al_2_O_3_) multilayer coatings. J. Alloy Compd..

[17] Michelakaki I., Bousoulas P., Maragos N., Boukos N., Tsoukalas D. (2017). Resistive memory multilayer structure with self-rectifying and forming free properties along with their modification by adding a hafnium nanoparticle midlayer. J. Vac. Sci. Technol. A Vac. Surf. Film.

[18] Wang H., Liu R., Jiang W.Q., Zhu J., Feng J.Z., Ding G.F., Zhao X. (2011). A novel method for improving the adhesion strength of the electrodeposited Ni films in MEMS. Appl. Surf. Sci..

[19] Suo X., Guo X., Li W., Planche M., Bolot R., Liao H., Coddet C. (2012). Preparation and characterization of magnesium coating deposited by cold spraying. J. Mater. Process Tech..

[20] Birringer R.P., Shaviv R., Geiss R.H., Read D.T., Dauskardt R.H. (2011). Effects of barrier composition and electroplating chemistry on adhesion and voiding in copper/dielectric diffusion barrier films. J. Appl. Phys..

[21] Huang R.X., Ma Z., Dong W.Z., Shen Y., Du F.M., Xu J., Jin M. (2019). On the Adhesive Strength Quantification and Tribological Performance of the Multilayered Fe–Ni Coating Fabricated by Electroplating. Strength Mater..

[22] Wang Y., Xu Z., Zhang A. (2019). Electrochemical dissolution behavior of Ti-45Al-2Mn-2Nb+0.8 vol% TiB_2_ XD alloy in NaCl and NaNO_3_ solutions. Corros. Sci..

